# The protective effect of procyanidin against LPS-induced acute gut injury by the regulations of oxidative state

**DOI:** 10.1186/s40064-016-3306-y

**Published:** 2016-09-22

**Authors:** Qiu Jue Wu, Yu Qin Wang, Yan Xia Qi

**Affiliations:** College of Animal Science and Technology, Henan University of Science and Technology, Luoyang, 471003 Henan People’s Republic of China

**Keywords:** Diamine oxidase, The morphology of the small intestine, Antioxidant, Procyanidins from pine needle, Broiler

## Abstract

**Background:**

A 2 × 4 factorial arrangement of treatments was used to investigate the protective effect of procyanidin (PCA) against lipopolysaccharide (LPS)-induced acute gut injury by the regulations of oxidative state for a 21-days feeding trial.

**Methods:**

A total of 384 1-days-old broiler chicks were assigned to 8 treatments with 8 replicate of 6 broiler chickens per pen. Broiler chickens fed diets based on 4 levels of dietary PCA (0, 0.05, 0.075 and 0.1 % of the requirements). Half of the birds from each treatment group were challenged with 0.9 % NaCl solution or LPS (250 μg/kg body weight, injection administered) at 16, 18 and 21 days of age.

**Results:**

The results indicated that, prior to LPS challenge, there was no dietary effect on bird growth performance (P > 0.05). The injection of LPS were also not associated with any significant changes in poultry performance (P > 0.05). But LPS injection increased serum diamine oxidase (DAO) level and the malondialdehyde (MDA) content of intestinal mucosa (P < 0.05), cause adverse effects to the morphology of the small intestine (P < 0.05), decreased the glutathione peroxidase (GSH-Px) and superoxide dismutase (SOD) activity of intestinal mucosa (P < 0.05). When LPS-challenged birds were pretreated with PCA, serum DAO concentration and MDA activity in jejunal and ileal mucosa were dramatically attenuated, and improved the morphology of the small intestine as well (P < 0.05).

**Conclusion:**

In conclusion, PCA is able to prevent LPS-induced oxidative stress response in vivo, improved the morphology of the small intestine. The beneficial effect of PCA may depend on increasing the activity of body’s antioxidant enzymes and scavenging free radical activity.

## Background

The animal gut is a natural environment for a dynamic microbial ecosystem associated with immune mediated disorders. Several disease (such as inflammatory bowel diseases, diarrheal and obesity) have been associated with changes in the composition of intestinal mucosal communities.

Chronic inflammation involves endotoxins derived from the gut flora. As a component of the Gram negative bacterial cell membrane, Lipopolysaccharide (LPS), is a potent biostimulant for lymphoreticular cells of the host’s immune system (Apicella et al. 1994), can induce immune dysfunction (Jiang et al. 2016). Animal are acutely sensitive to the toxic effects of LPS. Circulating endotoxins produced by gut microflora can induce immune hyperstimulation in the surrounding tissues and the production of cytokines by stimulating toll-like receptors.

It was established that the inflammatory response to LPS administration leads to increased release of reactive oxygen species, which alter the cytokine genes expression levels (Lund et al. 2006) and gut as active organ in immune system, is particularly vulnerable to the damage caused by free radicals (Mayssaa et al. 2015). Being one of major organs, intestine disruption of redox balance forced the development of similar changes in other internal organs (Błaszczyk et al. 2015). Thereby oxidative damage is considered to play a pivotal role in the occurrence of cancer and inflammation (Yen et al. 2008). So, much attention has been paid in search for antioxidants from natural sources to prevent oxidative damage.

Recently, traditional herbal medicines have attracted an increasing attention in the treatment of endotoxin injury, due to wide availability, multi-target action and low side effect (Ding et al. 2012). Pine needle, a famous Chinese medicine, has been widely used for the spasmolytic (Kar et al. 1975), anticancer (Singh et al. 2007), gastric antisecretory and antiulcer activities (Kumar et al. 2011), the antibrowning, antimicrobial and antioxidant activities (Zeng et al. 2014), anti-inflammatory effects (Zhang et al. 2009). Hsu et al. (2005) also noted that pine needle scavenges superoxide and inhibits the growth of leukemia cell. Apart from this, no reports regarding the biological effects of pine needle on an aviary animal model have been found so far.

Therefore, further investigation of PCA is warranted for a potential antioxidant and anti-inflammation source applied in new feed additive products. So, the aim of the present study was to investigate the effects of *E.Coli* lipopolysaccharide on the small immune mucosa and safety of PCA isolated from pine needle in animal model of acute inflammation and oxidative stress in broilers.

## Methods

### Broiler husbandry and experimental diets

All the experimental protocols were conducted so as to comply with specific guidelines approved by the Institutional Animal Care and Use Committee of Henan University of Science and Technology (Luoyang, People’s Republic of China). The study was conducted at a commercial broiler chicken farm (Distract Jianxi, Luoyang, Henan, China). The selected 384 1-day-old male broiler chicks (Arbor Acres) were obtained from a local hatchery (Xinan hatchery, Luoyang, Henan, China) and randomly divide into 8 treatments with 8 replicate pens of 6 broiler chickens per pen based on the initial body weight (P > 0.05). All the birds were housed in multi-tiered brooder cages placed in a climate-controlled room for the experimental period of 21 day. The house temperature was maintained at 35 °C during the first week, and it was reduced 2 °C per week until reaching the temperature of 24 ± 1 °C. Eighteen hours of lighting were provided per day throughout the duration of the experiment, apart from day 1 to 7 when 23 h of lighting were provided. The broiler chickens were provided access to feed and water ad libitum. Each pen was equipped with a separate feeder and a manual drinker.

The broiler chicks were all fed a proprietary starter ration. All experimental diets were formulated based on the National Research Council (1994) to meet or exceed the energy and nutrient requirements of the broiler. The basal diets (starter) were of the maize-soybean-type in Table 1 and provided as mash. The mixture was finally added to the basal diet and mixed in a vertical mixer for 5 min. Prepared feed was stored in a dry place to ensure the activity of probiotics.

**Table 1:** Ingredients and nutrient composition of the basal diet (g/kg diet as fed basis)

Ingredients (g/kg)	1–21 days
Corn	578
Soya bean meal (43 %, crude protein)	325
Corn gluten meal	30
Soybean oil	27
Limestone	9.5
Dicalcium phosphate	17.5
Salt	3
Choline chloride	3.0
Minerals premix^a^	2.5
Vitamin premix^b^	0.5
l-Lysine HCl	2.5
Methionine	1.5
Total	1000
Calculation of nutrients (g/kg)	
Apparent metabolism energy (MJ/kg)	12.5
Crude protein	212
Calcium	9.7
Available phosphorus	4.2
Lysine	10.8
Methionine	4.8
Methionine + cysteine	8.1

^a^The mineral premix provided the following per kg of diet: Fe (from ferrous sulfate), 80 mg; Cu (from copper sulfate), 8 mg; Mn (from manganese sulfate), 110 mg; Zn (Bacitracin Zn), 65 mg; iodine (from calcium iodate), 1.1 mg; Se (from sodium selenite), 0.3 mg

^b^Vitamin premix provided the following per kg of diet: Vitamin A (transretinyl acetate), 10,000 IU; Vitamin D_3_ (cholecalciferol), 3000 IU; Vitamin E (all-*rac*-*α*-tocopherolacetate), 30 IU; menadione, 1.3 mg; thiamine 2.2 mg; riboflavin, 8 mg; nicotinamide, 40 mg; pyridoxine·HCl, 4 mg; biotin, 0.04 mg; folic acid, 1 mg; vitamin B_12_ (cobalamine), 0.013 mg

The experiment used a completely randomized design with a 2 × 4 factorial arrangement of treatments, where main effects were abdominal injections of LPS challenge (with or without) and diet. Dietary treatment, which were the control group (birds fed with the basal diet); PCA (was provided by Shanghai Chaoxiang Biological Technology Co., Ltd., China. The purity was >95 %) groups (birds fed with the basal diet supplemented with 0.05, 0.075 and 0.1 % PCA), respectively. The LPS (*E. coli* serotype O55. B5; Sigma Chemical, St Louis, MO, USA) was dissolved in sterile 9 g/L (w/v) NaCl solution at 0.5 mg/mL, so that abdominal injections of 0.5 mL/kg body weight of solution would achieve the desired dosage. At 16, 18 and 21 days of age, half of the birds in each replicate were administered with LPS (250 μg/kg body weight) or an equivalent amount of sterile saline injection alone, which served as a control. Each replicate (8 birds) was the experimental units for dietary treatment and challenge status (injection administered LPS or saline).

Total daily feed intake/replicate were measured every morning at 07:00 from day 1 to 21 of age. Average daily feed intake was calculated at the end of each experimental period. Mortalities were recorded as they occurred and feed per gain values were corrected for mortality.

### Sample collection and processing

Body weight (BW) was measured at day 0, 14 and 21 of age to calculate average daily gain (ADG). Feed intake consumed per unit of body weight gain (F/G) was corrected for number of birds per pen and calculated on the basis of kg of feed consumed per kg of live BW gain. At the end of experimental period (21 day) six chicks from each treatment were randomly selected, and were sacrificed by bleeding from the jugular vein. Blood samples (around 5 mL each) were collected and centrifuged at 4450×*g* for 15 min at 4 °C to separate serum, which was frozen at −20 °C until further analysis. After blood collection, chickens were euthanized and necropsied. The abdominal cavity was opened and the small intestine was dissected free of the mesentery and placed on achilled stainless steel tray. The small intestine was divided into 3 segments: duodenum (from gizzard outlet to the end of the pancreatic loop), jejunum (from the pancreatic loop to Meckel’s diverticulum), and ileum (defined as the last two-thirds of the section between Meckel’s diverticulum and 2 cm anterior to the ileo-caeco-colonic junction, as described by Rodehutscord et al. (2012). The contents of the duodenum, jejunum, and ileum (aseptically) were flushed with ice-cold phosphate-buffered saline and the weight was recorded. After that, 5 cm segments were cut at the mid-jejunum and mid-ileum, respectively. The 5 cm intestinal segments were flushed gently with ice cold phosphate-buffered saline (pH 7.4) and then placed in 10 % fresh, chilled formalin solution for histological measurements. Approximately 20 cm of jejunal and ileal segments were opened longitudinally and mucosa were scratched carefully using a sterile glass microscope slide, respectively. Samples were collected into a 1.5 mL sterile tube and immediately frozen at −80 °C until further analysis. The pancreas, liver, spleen, gizzard and bursa of broiler chicks were also removed and weighed.

To avoid cross infection, samples from the unchallenged treatments were collected first. The challenged treatments were collected after the unchallenged sample collection had been completed.

### Intestinal morphology

Three cross-sections of the jejunum and ileum for morphological analysis were dehydrated and embedded in paraffin, sectioned at 5 μm thickness, and stained with hematoxylin and eosin. Villus height and crypt depth of twenty well-oriented villi per segment were measured using a Nikon ECLIPSE 80i light microscope equipped with a computer-assisted morphometric system (Nikon Corporation, Tokyo, Japan).

### Determination of the diamine oxidase (DAO) levels in the serum, mucosal antioxidant parameters

The levels of DAO in the serum were analyzed according to Li et al. (1996).

Approximately 0.3 g of jejunal and ileal mucosa samples were homogenised (1:9, wt/vol) with ice-cold 154 mmol/L sodium chloride solution using an Ultra-Turrax homogeniser (Tekmar Co., Cincinnati, OH) and then centrifuged at 4450×*g* for 15 min at 4 °C. The supernatant was then used for assaying mucosal antioxidant and immune parameters.

Total super-oxide dismutase (T-SOD), glutathione peroxidase (GSH-Px) and malondialdehyde (MDA) content were measured using diagnostic kits (Nanjing Jiancheng Bioengineering Institute, Nanjing, Jiangsu, P. R. China) according to the manufacturer’s instructions. The MDA concentrations were expressed as nmoL per mg of protein of mucosa tissue and units per milliliter for serum. Other enzyme activity was expressed as units per milligram of protein for tissues.

### Statistical analysis

The experiment was a 2 × 3 factorial arrangement, with dietary treatments and LPS challenge being the main factors. Statistical analyses were performed using the GLM procedure of statistical package for social sciences 18.0 (SPSS Inc., Chicago, IL, USA). The data were analyzed using multifactor analysis of variance (ANOVA) with treatment and challenge as factors. The differences between means were identified by the least significant difference (LSD). Differences among treatments and challenge were deemed to be significant only if the P value was less than 0.05.

## Results

### Growth performance

No significant differences were found in performance parameters of broilers among each treatment (P > 0.05) (Table 2). The interaction between PCA level and LPS challenge was not significant (P > 0.05) for performance parameters.

**Table 2:** Effects of PCA on the growth performance of LPS-induced oxidative stress broilers fed 1 to 21 days of age

Items	LPS (−)				LPS (+)				P values		
	Control	PCA1	PCA2	PCA3	Control	PCA1	PCA2	PCA3	Diet	Stress	Interaction
*1–15days*											
BWG, g/bird per d	58.5 ± 1.23	59.9 ± 2.40	59.6 ± 1.92	60.2 ± 2.07	57.3 ± 3.18	57.7 ± 1.34	57.3 ± 1.07	57.8 ± 1.91	0.577	0.825	0.981
FI, g bird per d	69.0 ± 2.41	69.9 ± 1.07	69.9 ± 2.37	70.0 ± 2.45	68.8 ± 1.21	68.7 ± 1.73	68.7 ± 1.68	68.8 ± 2.07	0.073	0.319	0.642
F/G	1.18 ± 0.12	1.17 ± 0.13	1.17 ± 0.08	1.16 ± 0.09	1.20 ± 0.10	1.19 ± 0.08	1.20 ± 0.07	1.19 ± 0.11	0.724	0.498	0.872
*15–21 days*											
BWG, g/bird per d	60.6 ± 2.04	61.0 ± 1.38	60.4 ± 1.28	60.9 ± 3.04	58.7 ± 1.27	58.8 ± 1.52	59.0 ± 2.34	59.3 ± 1.98	0.276	0.136	0.564
FI, g bird per d	98.8 ± 3.45	98.8 ± 3.27	98.7 ± 4.16	98.0 ± 2.97	96.8 ± 3.04	96.5 ± 2.64	96.1 ± 2.18	96.1 ± 3.06	0.796	0.145	0.921
F/G	1.63 ± 0.17	1.62 ± 0.15	1.63 ± 0.23	1.61 ± 0.09	1.65 ± 0.13	1.64 ± 0.06	1.63 ± 0.09	1.62 ± 0.10	0.816	0.381	0.577

Data represent means from 6 replicates per treatment

The P values represent the main effect of the diet, the main effect of LPS challenge and the interaction between the dietary treatments and LPS challenge

*BWG* Body weight gain, *FI* Feed intake; *F/G* feed intake consumed per unit of body weight gain; *LPS (−)* Dietary treatment were abdominal injections sterile saline (0.5 mg/kg body weigh); *LPS (+)* Dietary treatment were abdominal injections LPS (0.5 mg/kg body weigh). *Control* Basal diet; *PCA1* Basal diet supplemented with 0.05 % PCA; *PCA2* Basal diet supplemented with 0.075 % PCA; *PCA3* Basal diet supplemented with 0.1 % PCA

### Intestinal mucosal morphology

Compared with LPS unchallenged broilers, injection of LPS increased crypt depth but decreased the ratio of villus height to crypt depth in ileum (P < 0.05, Table 3), as well as reductions in the villus height and the ratio of villus height to crypt depth in jejunum (P < 0.05). The addition of PCA of the diet to oxidative stress broilers minimized the deleterious effects of LPS, improved the morphology of jejunum and ileum with or without injection administered LPS (P < 0.05), moreover, there have significantly difference on the morphology of jejunum and ileum (P < 0.05), and PCA is better for alleviating of LPS toxicity on the morphology of jejunum and ileum of broilers.

**Table 3:** Effects of dietary PCA on intestinal mucosal morphology of LPS-induced oxidative stress broilers fed 1 to 21 days age

Items	LPS (−)				LPS (+)				P values		
	Control	PCA1	PCA2	PCA3	Control	PCA1	PCA2	PCA3	Diet	Stress	Interaction
*Jejunum*											
Villus height (μm)	955. 7 ± 32.74^a^	1004.96 ± 40.21^b^	1049.53 ± 35.42^b^	1062.35 ± 30.74^b^	652.65 ± 20.34^c^	885.34 ± 24.37^a^	899.05 ± 26.37^a^	923.58 ± 25.37^a^	0.012	0.017	0.001
Crypt depth (μm)	153.00 ± 22.34	144.17 ± 20.41	148.62 ± 23.37	147.98 ± 21.03	183.91 ± 25.34	188.64 ± 26.41	186.30 ± 28.37	200.77 ± 29.31	0.045	0.023	0.035
Villus height : Crypt depth	6.25 ± 2.31^a^	6.97 ± 2.14^b^	7.06 ± 3.01^b^	7.18 ± 4.01^b^	3.55 ± 1.24^c^	4.69 ± 1.45^a^	4.83 ± 2.01^a^	4.60 ± 1.99^a^	0.011	0.020	0.018
*Ileum*											
Villus height(μm)	817.83 ± 21.11^a^	849.32 ± 22.03^b^	870.67 ± 24.13^b^	868.70 ± 20.19^b^	720.92 ± 19.42^a^	693.74 ± 20.31^a^	718.18 ± 21.17^a^	753.24 ± 18.64^a^	0.040	0.026	0.035
Crypt depth (μm)	131.09 ± 15.38^b^	128.47 ± 16.41^b^	125.06 ± 14.37^b^	126.42 ± 16.01^b^	168.47 ± 16.74^a^	158.35 ± 15.48^a^	146.02 ± 16.21^b^	142.74 ± 14.27^b^	0.006	0.004	0.572
Villus height : Crypt depth	6.24 ± 0.86^b^	6.61 ± 1.04^b^	6.96 ± 0.91^b^	6.87 ± 0.95^b^	4.28 ± 1.12^a^	4.38 ± 0.98^a^	4.92 ± 1.17^c^	5.28 ± 0.88^a^	0.009	0.031	0.766

Data represent means from 6 replicates per treatment

The P values represent the main effect of the diet, the main effect of LPS challenge and the interaction between the dietary treatments and LPS challenge

^a^, ^b^, ^c^ Least-square mean values (n 6) within a row with unlike superscript letters were significantly different (P < 0.05)

*LPS (−)* Dietary treatment were abdominal injections sterile saline (0.5 mg/kg body weigh); *LPS (+)* Dietary treatment were abdominal injections LPS (0.5 mg/kg body weight); *Control* Basal diet; *PCA1* Basal diet supplemented with 0.05 % PCA; *PCA2* Basal diet supplemented with 0.075 % PCA; *PCA3* Basal diet supplemented with 0.1 % PCA

### Serum parameters (DAO)

The results of DAO serum measurements are shown in Table 4. Before LPS challenge, there was no dietary effect on the activity of DAO (P > 0.05). LPS injection significantly increased the activity of DAO (P < 0.05). When LPS-challenged broilers were pretreated with PCA, these resulted in a decrease (P < 0.05) in the serum DAO level compared with the control groups (P < 0.05). The PCA interactions were also found to affect the DAO level in the serum.

**Table 4:** Effect of dietary PCA on serum parameters of LPS-induced oxidative stress broilers fed 1 to 21 days age

Items	LPS (−)				LPS (+)				P values		
	Control	PCA1	PCA2	PCA3	Control	PCA1	PCA2	PCA3	Diet	Stress	Interaction
DAO (U/ml)	16.51 ± 0.14^a^	15.42 ± 0.98^a^	15.40 ± 0.26^a^	15.18 ± 0.37^a^	30.52 ± 0.17^c^	22.45 ± 0.11^b^	20.81 ± 0.10^b^	19.34 ± 0.20^b^	0.002	0.001	0.020

Data represent means from 6 replicates per treatment

The P values represent the main effect of the diet, the main effect of LPS challenge and the interaction between the dietary treatments and LPS challenge

^a^, ^b^ Least-square mean values (n 6) within a row with unlike superscript letters were significantly different (P < 0.05)

*LPS (−)* Dietary treatment were abdominal injections sterile saline (0.5 mg/kg body weigh); *LPS (+)* Dietary treatment were abdominal injections LPS (0.5 mg/kg body weigh); *Control* Basal diet; *PCA1* Basal diet supplemented with 0.05 % PCA; *PCA2* Basal diet supplemented with 0.075 % PCA; *PCA3* Basal diet supplemented with 0.1 % PCA

### Intestinal antioxidant status

The results of the GSH-Px activities, SOD and concentrations of MDA in jejunum and ileal mucosa of chicks are presented in Table 5. Among the LPS-unchallenged groups, the enzyme activity of GSH-Px in jejunal and ileal mucosa was significantly increased (P < 0.05) in PCA3, compared to the CON treatment, PCA 2 and PCA3 treatments in chickens. Injection LPS significantly decreased the GSH-Px content in the jejunum mucosa of broilers (P < 0.05). Dietary supplementation with PCA had no influence on the GSH-Px activity in the jejunal and ileal mucosa of animals that were injection LPS (P < 0.05), and PCA did not recover the GSH-Px decrease induced by LPS in jejunum.

**Table 5:** Effects of dietary PCA on intestinal antioxidant and immune status of LPS-induced oxidative stress broilers fed 1 to 21 days age

Items	LPS (−)				LPS (+)				P values		
	Control	PCA1	PCA2	PCA3	Control	PCA1	PCA2	PCA3	Diet	Stress	Interaction
*Jejunum*											
SOD (U/mg pro)	48.01 ± 2.01^b^	48.84 ± 2.12^b^	49.53 ± 2.03^b^	56.12 ± 1.84^c^	41.73 ± 1.92^a^	45.99 ± 1.97^a^	46.87 ± 1.94^a^	47.03 ± 1.98^b^	0.001	0.001	0.054
GSH-Px (U/mg pro)	12.65 ± 1.57^b^	13.94 ± 1.24^b^	15.27 ± 1.42^b^	22.14 ± 1.15^c^	9.13 ± 1.20^a^	9.25 ± 1.04^a^	9.28 ± 0.98^a^	9.34 ± 0.87^a^	0.001	0.001	0.001
MDA (nmol/mg pro)	0.63 ± 0.12^a^	0.61 ± 0.14^a^	0.58 ± 0.09^a^	0.51 ± 0.14^a^	1.98 ± 0.17^c^	1.21 ± 0.20^b^	1.16 ± 0.21^b^	1.02 ± 0.14^b^	0.001	0.001	0.088
*Ileum*											
SOD (U/mg pro)	40.34 ± 1.76^b^	41.62 ± 2.13^b^	42.18 ± 1.82^b^	42.24 ± 2.07^b^	37.12 ± 1.95^a^	39.45 ± 2.34^a^	40.20 ± 1.64^b^	40.12 ± 2.21^b^	0.001	0.197	0.076
GSH-Px (U/mg pro)	10.77 ± 2.14^a^	11.26 ± 3.01^a^	17.13 ± 2.88^b^	19.94 ± 1.27^b^	10.21 ± 2.07^a^	10.57 ± 1.47^a^	11.85 ± 1.55^a^	12.21 ± 2.01^a^	0.001	0.061	0.054
MDA (nmol/mg pro)	0.37 ± 0.12^a^	0.32 ± 0.17^a^	0.30 ± 0.08^a^	0.29 ± 0.11^a^	0.46 ± 0.13^b^	0.39 ± 0.17^a^	0.34 ± 0.09^a^	0.30 ± 0.14^a^	0.001	0.001	0.051

Data represent means from 6 replicates per treatment

The P values represent the main effect of the diet, the main effect of LPS challenge and the interaction between the dietary treatments and LPS challenge

^a^, ^b^ Least-square mean values (n 6) within a row with unlike superscript letters were significantly different (P < 0.05)

*LPS (−)* Dietary treatment were abdominal injections sterile saline (0.5 mg/kg body weigh); *LPS (+)* Dietary treatment were abdominal injections LPS (0.5 mg/kg body weigh); *Control* Basal diet; *PCA1* Basal diet supplemented with 0.05 % PCA; *PCA2* Basal diet supplemented with 0.075 % PCA; *PCA3* Basal diet supplemented with 0.1 % PCA

Among the LPS-unchallenged groups, the SOD activity in the jejunal mucosa was increased in the PCA3 groups compared with the control group (P < 0.05). Dietary supplementation with PCA had no influence on the SOD activity in the ileal mucosa of animals that were not injection LPS (P < 0.05). Injection LPS significantly decreased the SOD activity in the jejunum and ileal mucosa of broilers (P < 0.05). Dietary supplementation with PCA3 had significant influence on the SOD activity in the jejunal and ileal mucosa of the LPS-treated broilers (P < 0.05), and PCA2 also significantly recovered SOD in ileum. The LPS-PCA interactions had no effect on the SOD activity in the jejunal and ileal mucosa.

The results presented in Table 5 showed the effects of each treatment on the MDA content in the intestinal mucosa. Among the LPS-unchallenged groups, the MDA content in the jejunal and ileal mucosa had no difference in the PCA groups compared with the control group (P > 0.05). Injection LPS significantly increased the MDA content in the intestinal mucosa of broilers (P < 0.05). Diet inclusion of PCA could significantly decreased the MDA content in the jejunal and ileal mucosa of broilers with LPS challenged, they have significantly attenuate the toxic effects of LPS (P < 0.05). The PCA-LPS interactions had no effect on the MDA content in the jejunal and ileal mucosa.

## Discussion

In recent years, the study of the anti-inflammatory and antioxidant effects of bioactive molecules presenting plant in animal has become more popular. How bioactive molecules influence the anti-inflammatory and antioxidant effects triggered during acute stress response remains to be fully understood. In the present study, proanthocyanidin (PCA), was used to assess their anti-inflammatory and antioxidant effects in a model of acute inflammation in broiler. To assess the anti-inflammatory and antioxidant effects of PCA, immune stress model of broiler for 21 days with the LPS from *E. coli* were performed. This model was chosen because it is enough to be caused a wide range of physiological responses, such as impaired intestinal morphology and function, compromised immunity and decreased antioxidant capacity which leads to feed utilization efficiency, retarded growth, as well as increased morbidity and mortality.

A previous study demonstrated that dietary supplementation with PCA may enhance detoxification of terpenes by providing precursors required for conjugation and excretion, thus ameliorating toxic effects (Foley et al. 1995). It might be predicted that supplementation with PCA could be more effectively and better absorbed by chicks during the infection and possibly ameliorate the adverse effects of LPS from *E.coli* infection on growth performance. This, however, in the present study, Before LPS challenge (0–15 days), there was no effect of dietary treatment on bird growth performance (P > 0.05). When LPS was administered injection, the body weight gain and feed intake of broilers were also not statistically significantly different from those given just saline (P > 0.05). FCR was not significantly affect by injection administration of LPS. The PCA-LPS interaction have no involve the production performance of broilers. From these results, we speculated that the efficiency of dietary PCA on the growth performance of broilers may be related to the additive dose of PCA and the environment conditions of the experimental house.

In the present study, no significant changes in poultry performance were found, but the morphology of the small intestine was significantly affected. The present study clearly showed that the acute response to LPS was only marginally influenced by dietary PCA. Compared with LPS unchallenged broilers, injection of LPS decreased the villus height, crypt depth and villus height/crypt depth of the submucosa of jejunum and ileum (P < 0.05). They have been attributed to irritant effects on the gastrointestinal tract (Awad et al. 2006a, 2006b). Small intestinal injury resulting from LPS use includes bleeding from ulcers and erosions, stricture or stenosis of the lumen, perforation of the wall, protein losing enteropathy, inhibition of cell division, and aggravation of preexisting inflammatory bowel diseases (Rocha et al. 2005). The pathogenesis of LPS-induced small intestinal injury is different from that of gastric injury. Under the oxidative stress conditions, oxidative stress due to mitochondrial dysfunction and inflammatory cascades play crucial roles in small intestinal mucosal injury (Konaka et al. 1999). Cellular oxidative stress results in disruption of epithelial integrity and translocation of bacteria (Cheung et al. 2014), thus leads to intestinal mucosal inflammation.

In fact, PCA not only protect against a LPS-induced damage but it has a direct effect on villi in the present study. They have been attributed to protective effects on the gastrointestinal tract. PCA have been recently reported to protect intestinal Caco-2 cell (Erlejman et al. 2006), and also shown to modulate intestinal cell functionality in vivo (Ramiro-Puig et al. 2008).

On the other hand, LPS can damage to lipids, lead lipid degradation and the superoxide radical and a biomarker of lipid degradation (MDA) are produced (Beckman et al. 1990). These free radicals and the superoxide anion destroy DNA and cause cellular damage, particularly injuring villous tip cells, causing functional and structural impairment of the brush border membrane (Basivireddy et al. 2002; 2003). But, the addition of PCA of the diet to oxidative stress broilers minimized the deleterious effects of LPS, improved the morphology of jejunum and ileum with or without injection administered LPS (P < 0.05), and suggested that PCA may be utilized by chicks during infection by reducing intestinal permeability, and could be used instead of antibiotics to maintain oxidative balance and immune defense against coccidian infection rather than for growth performance (Awad et al. 2006a).

The bioavailability of procyanidin in the gut system has been well documented in other studies (D’Archivio et al. 2007; Cheah et al. 2014). The unique polymerized structure of procyanidin can occur in the gut lumen and inhibits absorption across the small intestine, as they adhere to the gut mucosa, then protect the intestinal barrier (Manach et al. 2004; Cheah et al. 2014). In the current study, PCA were effective at maintaining villus height and crypt depth in the jejunal and ileal region, with most values approaching the values of healthy controls.

Furthermore, DAO in layer of villi of the intestinal mucosa reflects the function and structure of the small intestine and its plasma level can be used as a marker for evaluation of maturation and integrity of the intestinal mucosa. DAO activities or levels in serum are very low in normal conditions (Luk et al. 1980; Takagi et al. 1994), but when the intestinal mucosal epithelial cells are damaged, DAO will be released, and large amount of DAO will enter the systemic circulation (Song et al. 2009). In this study, the serum DAO levels of LPS model group were significantly increased compared with those in LPS (−) groups. Our results demonstrated that small intestinal mucosal permeability of the broilers with LPS was increased slightly. Therefore, the protective effect of PCA on the intestinal in the LPS broilers may be caused by reducing the intestinal mucosal permeability and/or improving the integrity of the intestinal mucosa. Moreover, the histomorphological change of jejunum and ileum mucosa was observed in the LPS broilers. These observations indicated that intestinal mucosa mechanical barrier of LPS broilers was impaired and intestinal permeability was increased. After administration of PCA, the ultrastructure of mucosal epithelial cell was improved, which demonstrates that PCA can repair the ultrastructure of the damaged cell, decrease the permeability, and inhibit inflammatory reaction, thus improving the intestinal mucosal barrier function.

PCA, a natural compound, are useful compounds that play a vital role as antioxidant function and free radical scavenger (Chen et al. 2014). In addition, such substances are believed to reduce oxidative stress in cells. In the present experiment, we observed a significant decrease in SOD and GSH-Px in LPS broilers. It is well showed that LPS stress has a lower antioxidant defense and enzymatic (SOD and GPx). Our observations are similar to the previous study (Li et al. 2015). On the other hand, PCA significantly increased antioxidant enzyme activity in LPS broilers. These results are in agreement with the studies of other authors (Kang et al. 2009; Samira et al. 2011).

Moreover, as a metabolite of biological membrane lipid peroxidation, the levels of MDA can reflect the status of oxidative stress. In our data suggested that LPS could enhance intestinal lipid peroxidation by inhibiting antioxidant enzymes. However, co-treatment with PCA abolished LPS-induced lipid peroxidation, and we observed that the levels of MDA in the LPS + PCA treated groups were significantly decreased compared to those of the LPS group. The activities of SOD in the LPS + PCA groups were significantly increased compared to those of the LPS group. Therefore, PCA should be protective against small intestinal mucosal injury in which free radical production and tissue antioxidant depletion play crucial roles in cell and tissue damage. This protective effect of PCA is suggestive to be related to an increasing the activity of body’s antioxidant enzymes and scavenging free radical activity (Chen et al. 2013).

## Conclusions

In summary, results presented in the current paper show that procyanidins, was favorable for chickens, especially in the presence of stress, which can exerts potent in vivo antioxidant effects in chickens by increasing the activity of body’s antioxidant enzymes and scavenging free radical activity. Hence, the present study offers new perspectives for the treatment of oxidative stress in young broilers. Further studies are required to explain the mechanism of action of PCA toward oxidative stress.
